# Clinical outcomes of peripartum cardiomyopathy: a 15-year nationwide population-based study in Asia

**DOI:** 10.1097/MD.0000000000008374

**Published:** 2017-10-27

**Authors:** Victor Chien-Chia Wu, Tien-Hsing Chen, Jih-Kai Yeh, Michael Wu, Cheng-Hui Lu, Shao-Wei Chen, Katie Pei-Hsuan Wu, Chun-Wen Cheng, Chih-Hsiang Chang, Kuo-Chun Hung, Ming-Shyan Chern, Fen-Chiung Lin, Ming-Shien Wen

**Affiliations:** a Division of Cardiology, Chang Gung Memorial Hospital, Linkou Medical Center, Taoyuan City; b Department of Cardiology, Chang Gung Memorial Hospital, Keelung; c Divison of Cardiology, Weill Cornell Medical Center, New York, NY; d Department of Cardiothoracic and Vascular Surgery, Chang Gung Memorial Hospital, Linkou Medical Center, Taoyuan City; e Department of Rehabilitation, Chang Gung Memorial Hospital, Linkou Medical Center, Taoyuan City; f Department of Infectious Diseases, Chang Gung Memorial Hospital, Linkou Medical Center, Taoyuan City; g Department of Nephrology, Chang Gung Memorial Hospital, Linkou Medical Center, Taoyuan City; h College of Medicine, Chang Gung University, Taoyuan City, Taiwan.

**Keywords:** epidemiology, outcome, peripartum cardiomyopathy

## Abstract

Peripartum cardiomyopathy (PPCM) is the development of heart failure during late pregnancy to months postpartum with potential fatal outcome. However, the disease is not well-studied in Asia.

We aimed to investigate the epidemiology and clinical outcomes of PPCM in Taiwan.

Electronic medical records were retrieved from Taiwan National Health Insurance Research Database from 1997 to 2011. Patients with PPCM were separated into 3 groups based on the timing of diagnosis. Early: PPCM diagnosed first to ninth month of pregnancy. Traditional: PPCM diagnosed last month of pregnancy till fifth month post-delivery. Late: PPCM diagnosed sixth to twelfth month post-delivery. Primary outcomes defined as cardiac death, all-cause mortality, and major adverse cardiovascular events (MACE) within 1 year.

A total of 3,506,081 deliveries during 1997 to 2011 were retrieved and 925 patients with PPCM were identified. Overall incidence of PPCM was 1:3,790 during the 15 years. Early, Traditional, and Late group each had 88, 742, and 95 patients. Cardiac death occurred in 31 patients, all-cause mortality in 72 patients, and MACE in 65 patients. Late group had 2- to 3-fold event rates in cardiac death, all-cause mortality, and MACE compared with Early and Traditional groups. Cumulative incidence showed significant differences for cardiac death (*P* = .0011), all-cause mortality (*P* = .0031), and MACE (*P* = .0014) among 3 groups. Multivariate Cox model showed Late group had significantly worse outcomes after adjusted for clinical variables compared with 2 other groups.

Our study is the largest national cohort among Asian countries that showed timing of diagnosis of PPCM had different outcomes. Late diagnosis portended significantly increased morbidity and mortality, even after adjusted for clinical variables.

## Abstract 2

Supplemental Digital Content is available in the text

## 1 Introduction

Peripartum cardiomyopathy (PPCM) is a rare cause of heart failure (HF) in pregnant women at the time of or following childbirth that is potentially fatal. PPCM is diagnosed when the following criteria defined by National Heart, Lung, and Blood Institute in 1971^[1,2]^ and the Office of Rare Diseases Research in 1997^[3,4]^ are met: development of HF in the last month of pregnancy or within 5 months of delivery; absence of a determinable etiology for HF; absence of demonstrable heart disease before last month of pregnancy; and echocardiographic evidence of left ventricular (LV) systolic dysfunction. A broader description of PPCM was given by Heart Failure Association of the European Society of Cardiology Working Group as an idiopathic cardiomyopathy presenting with HF secondary to LV systolic dysfunction toward the end of pregnancy or in the months following delivery, where no other cause of HF is found.^[5]^

Etiology of PPCM has been attributed to viral myocarditis, autoimmune response, abnormal hemodynamic response, hormonal abnormality, malnutrition, genetic mutation, and dilated cardiomyopathy.^[6–8]^ The national incidences of PPCM ranged from 1 in 300 live births in Haiti^[9]^ to a mean of 1 in 3,189 live births from US National Hospital Discharge Survey.^[10]^ Using Southern California health care registry, ethnic incidences of PPCM in whites, African-Americans, Hispanics, and Asian-Americans were 1 in 4,075, 1 in 1,421, 1 in 9,861, and 1 in 2,675 deliveries, respectively.^[11]^ However, current literatures for PPCM among Asian countries remain scarce.^[12,13]^

Though most PPCM were diagnosed in the first week post-delivery, there were pregnancy-associated HFs occurring months prior to delivery to months post-delivery. Using the information from 15-year cohort of women with deliveries provided by Taiwan National Health Insurance Research Database (NHIRD), we aim to study the epidemiology of PPCM in Taiwan, and compare the outcome of broadly defined HF occurring early during pregnancy and HF occurring late up to 1 year following delivery that is considered postpartum period,^[14–16]^ to traditionally defined PPCM with HF occurring during last month of pregnancy to 5 months after delivery.

## 2 Methods

### 2.1 Study patients

Taiwan NHIRD started in 1995 and provides 99.5% coverage for the 23 million residents in Taiwanese.^[17]^ The database provides all dates of inpatient and outpatient services, diagnosis, prescriptions, examinations, operations, and expenditures, and data are updated biannually. Institutional Review Board of CGMH approved this study.

By searching medical records from NHIRD, we retrieved all women hospitalized with HF from 10 months prior to the delivery till 12 months postpartum between January 1, 1997 and December 31, 2011. HF was initially screened by International Classification of Diseases, 9th Revision, Clinical Modification (ICD-9-CM) codes HF (428.xx), primary and secondary cardiomyopathies (425.4, 425.9), PPCM (674.5), and myocarditis (429.0) (Appendix). A detailed review of medical history was done to confirm the diagnosis of PPCM if all of the following criteria were satisfied: no previous diagnosis of HF, diagnosis of HF occurring during up to 10 months prior to delivery till 12 months post-delivery, and no other cause of HF could be identified. This definition fulfills the broadly defined criteria by ESC Working Group.

One limitation of NHIRD is that detailed report of an examination such as ejection fraction are not provided in the retrievable database, therefore diagnoses using ICD-9-CM code in NHIRD were previously validated against the gold standard hospital electronic medical records (EMR) with high accuracy. For instance, diagnosis of hypertension in NHIRD had 97% sensitivity and 95% positive predictive value (PPV) against hypertension in EMR, with diabetes had 98% sensitivity and 95% PPV, and HF had 99% sensitivity and 99% PPV.^[18]^ We further categorized PPCM patients into 3 groups. Early group: PPCM diagnosed as early as first month till ninth month of pregnancy. Traditional group: PPCM diagnosed as previously defined in the last month of pregnancy till fifth month post-delivery. Late group: PPCM diagnosed from sixth month till twelfth month post-delivery.

### 2.2 Covariate and study outcomes

Previous literature suggested clinical variables such as maternal age, history of preeclampsia/eclampsia, hypertension, gestational diabetes, diabetes mellitus (DM), multiple pregnancy, number of children born, and race are associated with outcome prognosis.^[6,7]^ With over 95% of Taiwan's 23 million population consisting of Han Chinese, our study was of uniform ethnic background. The clinical characteristics of our study patients were significantly different among 3 groups in previous delivery, delivery type at PPCM, multiparity at PPCM. Therefore in multivariate analysis we entered these known factors such as age, previous delivery, delivery type at PPCM, multiparity at PPCM, history of preeclampsia/eclampsia or hypertension, history of gestational diabetes, or DM as covariates to determine outcome difference among groups.

The medical records of NHIRD listed primary diagnoses of the patients during admission. Definitions of cardiovascular death meet the criteria of Standardized Definitions for End Point Events in Cardiovascular Trials draft by the Food and Drug Administration. Death and causes of death were retrieved according to registry data of NHIRD. Primary outcomes were cardiac death, all-cause mortality, and major adverse cardiovascular events (MACE), including acute myocardial infarction, cerebrovascular accident, HF readmission, heart transplant, and cardiac death.

### 2.3 Statistical analysis

Patients’ clinical characteristics among the study groups were compared using Fisher exact test for categorical variable and one-way analysis of variance for continuous variable. Cumulative incidence function was plotted for cardiac death, all-cause mortality, and MACE within 1 year among study groups, and log-rank test was performed to compare group difference. Multivariable Cox proportional hazard models were performed on time to event for cardiac death, all-cause mortality, and MACE within 1 year among study groups, adjusted for age, previous delivery, delivery type at PPCM, multiparity at PPCM, history of preeclampsia/eclampsia or hypertension, and history of gestational diabetes or DM. All statistical analyses were carried out using commercial software (SPSS, version 22, IBM, Armonk, NY).

## 3 Results

### 3.1 Incidence

**Figure 1:**
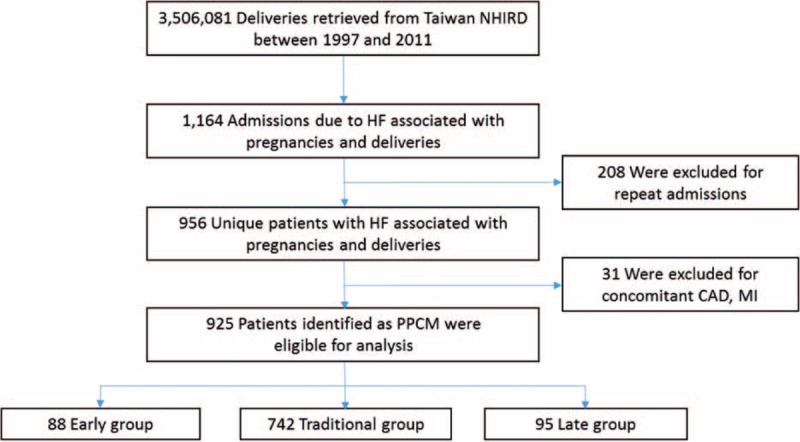
Study design and screening criteria flow chart for the inclusion of peripartum cardiomyopathy (PPCM) study patients. CAD = coronary artery disease, MI = myocardial infarction.

**Figure 2:**
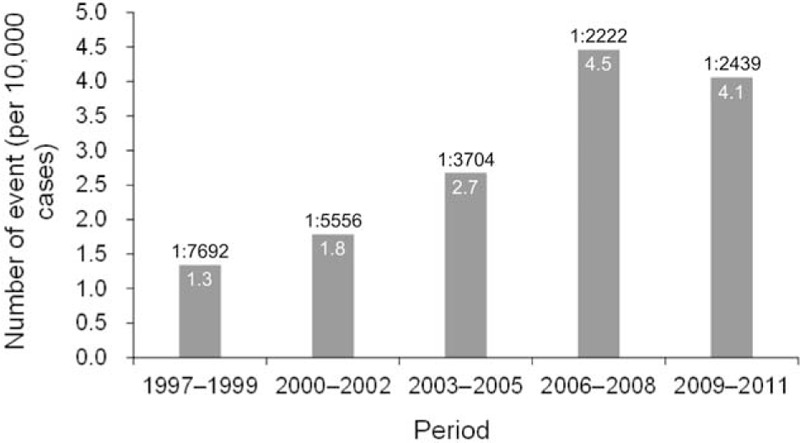
Incidence of peripartum cardiomyopathy (PPCM) between 1997 and 2011. Numbers above bars are incidence per 3-y interval. Numbers within the bars are number of cases of PPCM per 10,000 deliveries.

**Figure 3:**
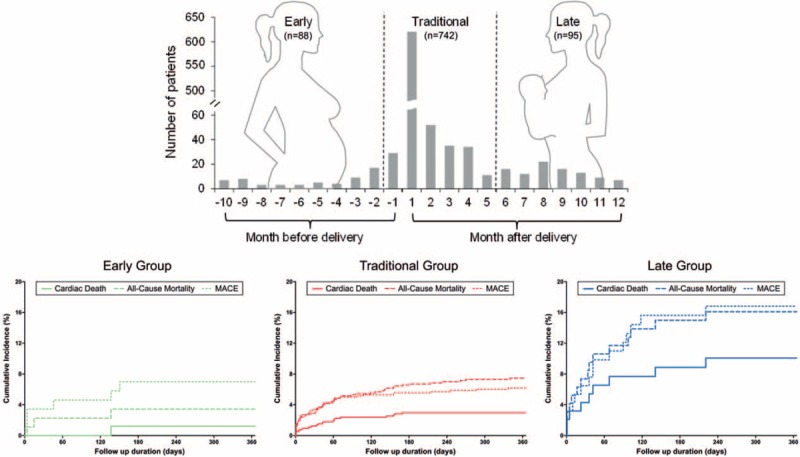
Upper panel: Timing of diagnosis and outcomes of PPCM in the 925 study patients. Lower panel: One-year cumulative incidence for cardiac death, all-cause mortality, and major cardiovascular events (MACE) by Early group (diagnosed first to ninth month of pregnancy), Traditional group (diagnosed last month of pregnancy till fifth month post-delivery), and Late group (diagnosed sixth to twelfth month post-delivery). Late group was associated with overall higher incidence of cardiac death, all-cause mortality, and MACE compared with 2 other groups.

A total of 3,506,081 deliveries were retrieved from NHIRD between 1997 and 2011. A total of 1,164 admissions with diagnosis of HF associated with pregnancy and delivery were found. After excluding 208 repeated admissions and excluded 31 patients with concomitant coronary artery disease and myocardial infarction, a final of population of 925 patients (mean age 30.4 ± 5.7) with PPCM were identified (Fig. 1), with mean follow-up of 5.4 ± 4.1 years. The incidence of PPCM was 1 in 3,790 deliveries during the 15-year span, with increased incidence per 3-year interval from 1997 to 2008 and decreased from 2009 to 2011 (Fig. 2). There were 88 patients in the Early group, 742 patients in the Traditional group, and 95 patients in the Late group (Fig. 3, upper panel). Most of the diagnosis of PPCM fell within the traditionally defined period, with the peak of the number of diagnosis within 1 month of delivery (610/925 = 65.9%).

### 3.2 Study patients

Clinical characteristics of the patients are listed in Table 1. In reviewing delivery history of the patients in 3 groups, there was significant difference in previous delivery (*P* = .046), delivery types (abortion, vaginal delivery, and Cesarean section) at diagnosis of PPCM (*P* < .001), and multiparity at diagnosis of PPCM (*P* = .003) among groups (Table 1). In reviewing medical history of the patients, there was significant difference in preeclampsia/eclampsia among 3 groups (*P* = .049), but there were no differences in hypertension, gestational diabetes, DM, and hyperlipidemia. In terms of management (Table 2), there was significant difference in patients receiving cardiac catheterization (*P* = .003), but there were no differences in patients receiving intra-aortic balloon pump (IABP) or extracorporeal membrane oxygenation (ECMO). Patients in Traditional and Late groups were more likely to receive angiotensin converting enzyme inhibitor (ACEi) or angiotensin receptor blocker (ARB), beta-blockers, diuretics, spironolactone, and digoxin for HF treatment and inotropic agents for HF with decompensation. PPCM-targeted drug bromocriptine was used in a number of patients within Traditional group. Patients in Traditional group who had a higher percentage stayed in ICU, with longer ICU days and higher in-hospital death.

**Table 1:**
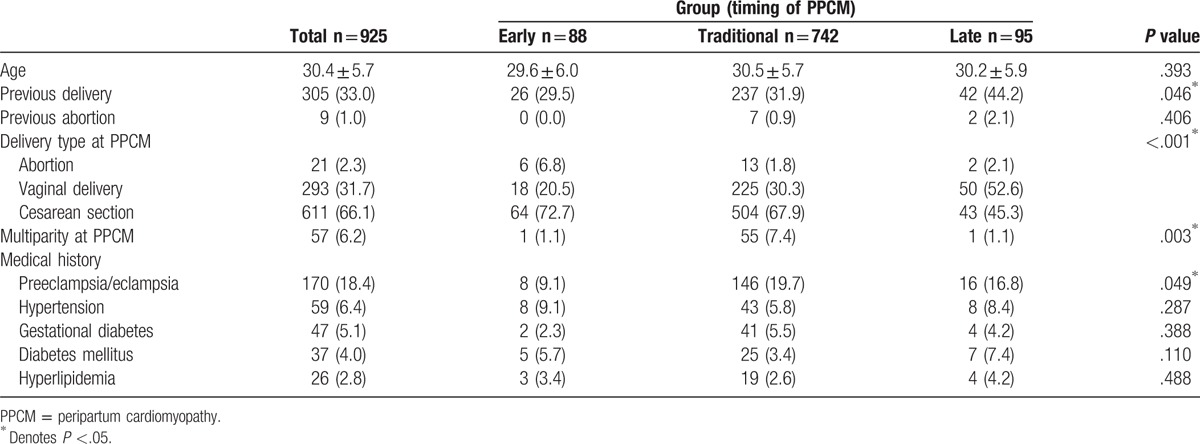
Clinical characteristics of the study patients.

**Table 2:**
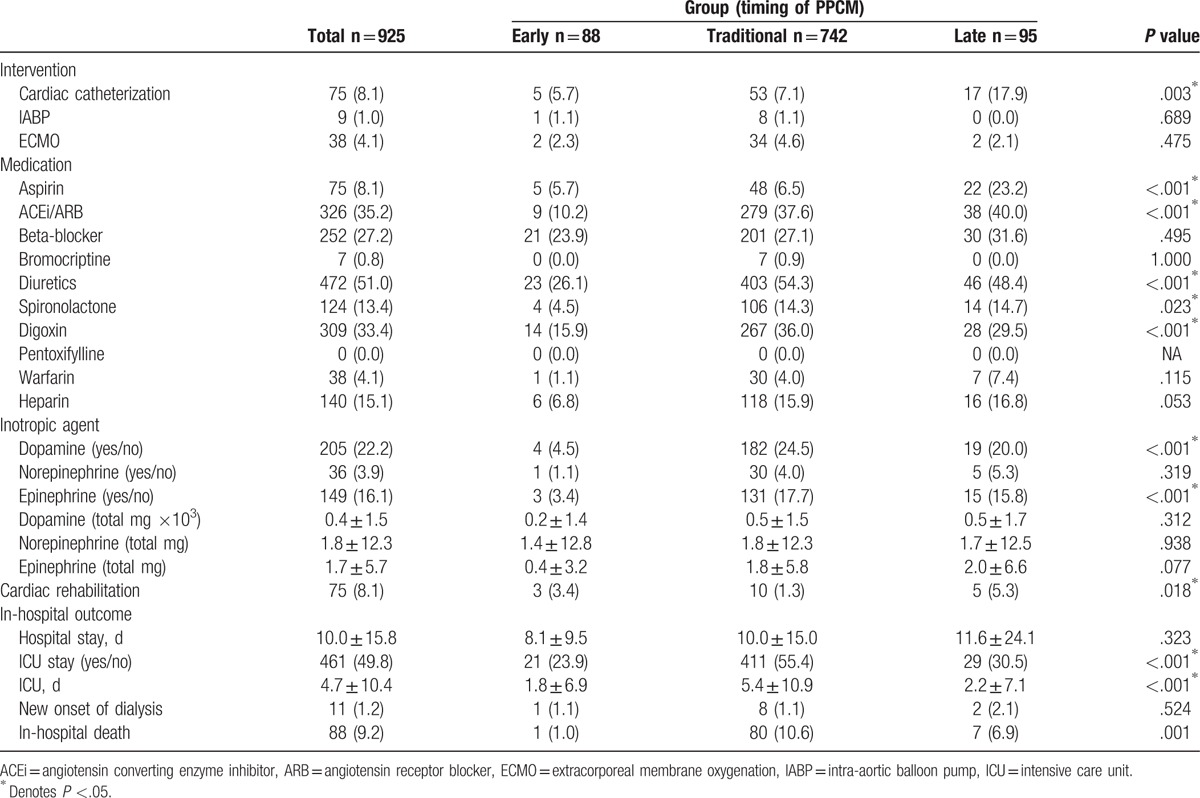
Management and outcome in the study patients.

### 3.3 One-year outcome

Primary outcomes of cardiac death occurred in 31 patients, all-cause mortality in 72 patients, and MACE in 65 patients within 1 year. There were 4 patients in Traditional group and 1 patient in Late group who underwent cardiac transplantation. Overall, patients in Late group had worse prognosis compared with pregnancy-associated HF in Early group and previously defined PPCM in Traditional group (Fig. 3, lower panel), with higher rate of cardiac death (9.5% vs 1.1% and 2.8%), all-cause mortality (15.8% vs 3.4% and 7.3%), and MACE (15.8% vs 6.8% and 5.9%) (Table 3).

**Table 3:**
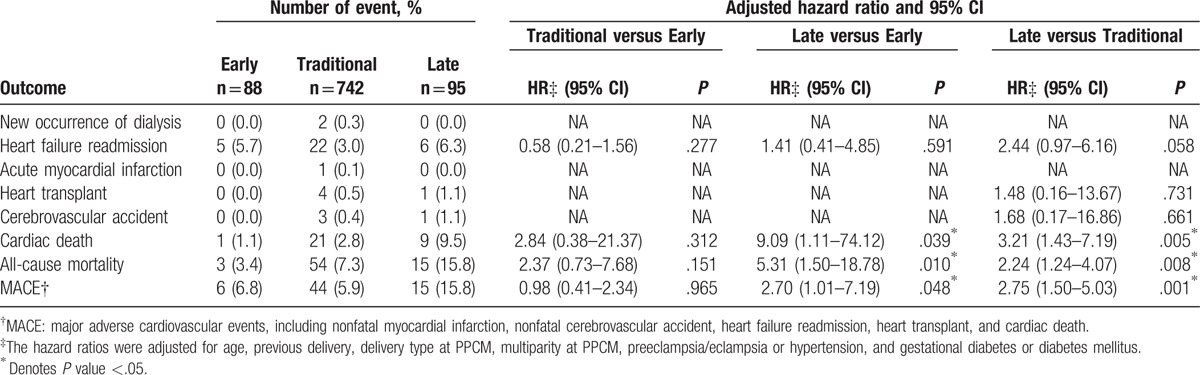
One-year outcome by timing of diagnosis for peripartum cardiomyopathy.

**Figure 4:**
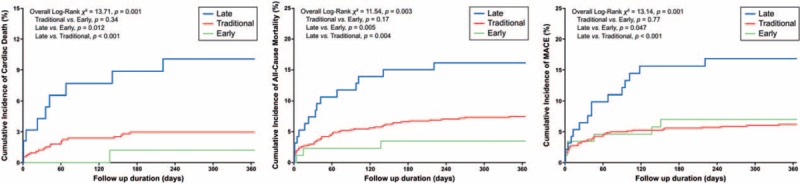
One-year cumulative incidence for diagnosis groups by cardiac death, all-cause mortality, and MACE.

Cumulative incidence plots showed significant differences among 3 groups for all primary outcomes: cardiovascular death (*P* = .001), all-cause mortality (*P* = .003), and MACE (*P* = .001). In terms of cardiac death, patients in Late group had significantly worse prognosis compared with Early group (*P* = .012) and Traditional group (*P* < .001). In terms of all-cause mortality, patients in Late group also had significantly worse prognosis compared with Early group (*P* = .005) and Traditional group (*P* = .004). In terms of MACE, patients in Late group again had significantly worse prognosis compared with Early group (*P* = .047) and Traditional group (*P* < .001). There was no difference in cumulative events between Early and Traditional groups for all primary outcomes (Fig. 4).

In multivariate Cox proportional hazards models, clinical variables of age, previous delivery, delivery type at PPCM, multiparity type at PPCM, preeclampsia/eclampsia or hypertension, and gestational diabetes or DM were adjusted (Table 3). With regard to cardiac death, patients in Late group had significantly worse outcome compared with Early group (hazards ratio [HR] = 9.09, CI: 1.11–74.12, *P* = .039) and Traditional group (HR = 3.21, CI: 1.43–7.19, *P* = .005). With regard to all-cause mortality, patients in Late group also had significantly worse outcome compared with Early group (HR = 5.31, CI: 1.50–18.78, *P* = .010) and Traditional group (HR = 2.24, CI: 1.24–4.07, *P* = .008). With regard to MACE, patients in Late group again had significantly worse outcome compared with Early group (HR = 2.70, CI: 1.01–7.19, *P* = .048) and Traditional group (HR = 2.75, CI: 1.50–5.03, *P* = .001). There was no difference between Early and Traditional groups with regard to cardiac death, all-cause mortality, or MACE.

## 4 Discussion

Our study had several findings. This is the largest national cohort study of PPCM in Asia. The incidence of PPCM in Taiwan in recent years was comparable to Asian Americans in the United States, suggesting genetic underpinnings have an important role in PPCM. A majority of the PPCM occurred within the first month following delivery, suggesting peripartum stress is the most likely underlying mechanism for causing PPCM. A new category, the Late group, defined as PPCM diagnosed sixth to twelfth months post-delivery, showed significantly worse clinical outcome compared with both Early group and Traditional group.

### 4.1 Epidemiology of PPCM

Currently, published studies of PPCM were from South Africa, Haiti, Brazil, Germany, Japan, and United States with prevalence from 1:300 in Haiti to 1:20,000 in Japan^[9–11,19–23]^ and data in other countries have been lacking. Our study used nationwide health insurance program in Taiwan, allowing the study of information on incidence, maternal cardiovascular and delivery history, associations, interventions, medications, and 1-year outcome of PPCM without selection and participation biases. The epidemiology of PPCM in Taiwan during the 15-year was 1 in 3,790, which was higher than previously reported incidence in Japan but similar to Asian Americans with 1 in 2,675.^[11]^

Most previous studies enrolled patients with pregnancy-associated HF from last trimester prior to delivery till 5 months post-delivery. Our study is unique in that patients with myocardial failures occurred from early pregnancy till extended months post-delivery were included for analysis, and there were quite a number of patients in the Early and Late groups with unexplained HF. With most of patients diagnosed of PPCM in the first month following delivery at 65.9%, peripartum stress due to altered physiological conditions was the most important cause of the condition.

### 4.2 Associated conditions, treatment, and outcome of PPCM

From the clinical characteristics of study patients, a higher incidence of previous delivery was found in Late group (44.2%) compared with Early (29.5%) and Traditional (31.9%) groups. This suggests previous births may delay the onset, presentation, and diagnosis of PPCM. There was also higher percentages of vaginal delivery in Late group (52.6%), compared with Early (20.5%) and Traditional (30.3%) groups, suggesting higher stability during delivery and possibly more indolent presentation of the PPCM in the Late group. On the other hand, a higher rate of Cesarean sections higher in Early (72.7%) and Traditional (67.9%) groups compared with the Late group (45.3%) may suggest increased maternal risks requiring immediate delivery. Risk factors speculated for the development of PPCM are advanced maternal age, high number of parity, high number of gravidity, twin pregnancy, use of tocolytic therapy, African descent, non-Caucasian ethnicity, and poverty.^[24]^ Multiparity has been traditionally considered a risk factor for PPCM, however most studies in the United States have reported the development of PPCM in conjunction with the first or second pregnancy in 50% of patients.^[6]^ Previous study noted rate of multiple birth in PPCM was 9%, whereas the rate in average estimate was 3%.^[25,26]^ In our study, the rate of multigestations in Traditional group was 7.4%.

In PPCM, oxidative stress plays a central part in disease pathogenesis. The vasculo-hormonal hypothesis was tested in experimental study, and STAT3 was shown to play a role in cardiomyocyte protection from reactive oxygen species (ROS). Loss of STAT3 in murine model leads to increased ROS, triggering secretion of cathepsin D that in turn cleaves prolactin into 16-kDa fragment promoting cell death in PPCM.^[27]^ Bromocriptine blocks prolactin secretion and was effective in the treatment of PPCM in mice. Prospective observation registry in Germany has shown promise of 4-week bromocriptine therapy on top of standard HF medications including beta-blockers and ACEi/ARB, with higher recovery rate.^[19]^ In our study, bromocriptine was used in 7 patients (0.9%) in Traditional group but none in Early and Late groups.

Patients with PPCM at times present with rapid progression leading to critical LV failure and acute pulmonary edema, requiring use of inotropic agents and mechanical assist device such as IABP and ECMO. Inotropes was used more often in Traditional and Late groups, possibly reflecting the more severely depressed LV function in these patients than Early group. Cardiac rehabilitation has been shown to improve clinical status, HF readmission, and outcome. This exercise training was prescribed with higher rates in Early and Late groups compared with Traditional group, reflecting the higher exercise capacity in the early pregnancy or extended months post-delivery.

A national inpatient database revealed in-hospital mortality in United States during 2004 to 2011 was 1.3% for patients with PPCM.^[23]^ We reported an overall in-hospital death of 9.2% in these women with PPCM, and 1.0%, 10.6%, and 6.9% for Early, Traditional, and Late groups, respectively. Recently, IPAC study reported that the mortality rate was 4% in the 100 women with PPCM that were followed up 1-year post-partum.^[28]^ In our 1-year follow-up, cardiac death was found in 1.1%, 2.8%, and 9.5% whereas all-cause mortality was 3.4%, 7.3%, and 15.8% in Early, Traditional, and Late groups, respectively.

### 4.3 The late group

In the study by Elkayam et al,^[29]^ the authors noted classic criteria for the diagnosis of PPCM as established by Demakis et al^[1,2]^ limited the diagnosis to the last gestational month and first 5 months after delivery. However, several reports published later described women presented with cardiomyopathy earlier in the pregnancy.^[30–34]^ In addition, although PPCM is usually diagnosed within the first 5 months postpartum, it is often missed or delayed because most of the signs and symptoms of normal pregnancy are similar to those of HF.^[35]^ In addition, pregnancy-associated cardiovascular death can occur up to 1 year following delivery.^[16]^ Furthermore, PPCM and pregnancy-associated cardiomyopathy had been described to be part of the same clinical spectrum.^[29]^

We included the patients with HF occurring >5 months post-delivery in the Late group since hormonal imbalance in postpartum women can persist as long as 12 months after delivery with some experts considering postpartum care necessary up to 1 year after giving birth.^[13,14]^ Importantly, there were noticeable differences in outcomes of PPCM between the study groups. Both cumulative incidence and multivariate Cox proportional hazards model showed significantly worse prognosis in all primary outcomes in Late group, whereas no difference existed between Traditional and Early group. The 1-year rate of cardiac death, all-cause mortality and MACE in Late group was more than 2 to 3 times compared with Early and Traditional groups.

As stated earlier, the genetic evidence in the development of PPCM was demonstrated in the animal model with loss of STAT3, leading to increased ROS, cleaving of prolactin into 16-kDa fragment, and eventual cardiomyocyte apoptosis. Through negative-feedback loop, prolactin secretion is typically regulated and inhibited by dopamine.^[36]^ With secretion of placental lactogen, relatively low levels of prolactin is maintained during early and mid-pregnancy.^[36]^ In combination with reduction of dopamine secretion and insensitivity to the negative feedback mechanism, the effects of placental lactogen is overcome with the large nocturnal surge of prolactin prior to parturition.^[36]^ The prolactin level then remains elevated while breastfeeding continues during postpartum.^[37,38]^ This period typically lasts 1 year as guideline recommendations^[39]^ and may coincide with the late onset of PPCM. On the other hand, a study also reported that breastfeeding had no adverse effects to the mother in 67% of patients with PPCM, and instead was associated with recovery of LV systolic function.^[40]^

Hypertensive pregnancy disorders complicated 5% to 7% of all pregnancies, and been linked to later cardiovascular events.^[41]^ Preeclampsia has also been shown to be associated with persistent postpartum cardiovascular impairment and abnormal LV function in prospective longitudinal case-control study.^[42]^ Our study patients had a higher percentage of preeclampsia/eclampsia or hypertension in Traditional group (25.5%) and Late group (25.2%) compared with Early group (18.2%). The combination of aforementioned higher incidence of prior births, genetic mutation, postpartum hormonal imbalance, especially elevated prolactin level, and preeclampsia/eclampsia or hypertension in the Late group may explain the worst outcome among all patients. In summary, our findings showed that late presentation and diagnosis of PPCM had distinctly higher cardiac death, all-cause mortality, and MACE.

## 5 Limitations

There are several limitations in epidemiologic data from NHIRD. First, using ICD-9-CM codes for patient screening may miss some cases for conditions not coded correctly. Second, the main criteria used in diagnosis of PPCM using LV ejection fraction was not available. However as mentioned in the Methods section, the diagnosis of HF by NHIRD has high accuracy against the gold standard EMR. Third, the diagnosis of HF associated with pregnancy, delivery, and postpartum period required the patients to have delivery at hospital inpatient services. In rare situations patients may still give birth at clinics not using ICD codes. Last, since our study consisted of uniform ethnic background, and application of the results to other populations awaits further studies.

## 6 Conclusions

Our study of PPCM was the largest nationwide population-based cohort in Asia that showed timing of diagnosis of PPCM had different outcomes. Late group of patients with PPCM had significantly worse outcome compared with both Early and Traditional groups, even after adjusted for clinical variables.

## Supplementary Material

Supplemental Digital Content
