# Supplementary material for: Clinical outcomes of peripartum cardiomyopathy: a 15-year nationwide population-based study in Asia
Source: Medicine (Baltimore). 2017 Oct 27;96(43):e8374. doi: 10.1097/MD.0000000000008374 (PMC5671863; doi:10.1097/MD.0000000000008374)
Supplement: Supplemental Digital Content [file medi-96-e8374-s001.docx]

Appendix. ICD-9-CM code used for diagnosis in the current study

| Variable | Code |
| --- | --- |
| Heart failure | 428.xx (for enrollment) |
| Cardiomyopathies | 425.4, 425.9 (for enrollment) |
| Peripartum cardiomyopathy | 674.5 (for enrollment) |
| Myocarditis | 429.0x (for enrollment) |
| Coronary artery disease | 410.xx–414.xx (for exclusion) |
| Myocardial infarction | 410.xx, 412.xx (for exclusion) |
| Congenital heart disease | 745.xx–747.xx (with catastrophic illness certificate) |
| Hypertrophic cardiomyopathy | 425.1x |
| Sepsis | 038.xx |
| Preeclampsia/eclampsia | 642.4x–642.7x |
| Gestational diabetes mellitus | 648.0x–648.8x |
| Diabetes mellitus | 250.xx |
| Hypertension | 401.xx–405.xx |
| Dyslipidemia | 272.xx |
| Cardiovascular death |  |
| Acute myocardial infarction | 410.xx |
| Sudden cardiac death | 427.5 |
| Heart failure | 428.xx |
| Cardiogenic shock | 785.51 |
| Any stroke | 430.xx–437.xx |
| Dysrhythmia | 427.xx |
| Pulmonary embolism | 415.1, 415.11, 415.19 |
| Aortic aneurysm dissection | 441.xx |
| Peripheral arterial disease | 443.9 |
